# Maternal–Fetal Crosstalk in Cardiovascular Programming: Linking the Intrauterine Environment to Lifelong Disease Risk

**DOI:** 10.3390/jcdd13070292

**Published:** 2026-06-24

**Authors:** Ning Wu, Hairui Sun, Siyao Zhang, Jiaqi Fan, Tong Yi, Ruimin Liu, Yihua He

**Affiliations:** 1 Echocardiography Medical Center, Beijing Anzhen Hospital, Capital Medical University, Beijing 100029, China; 2 Maternal-Fetal Medicine Center in Fetal Heart Disease, Beijing Anzhen Hospital, Beijing 100029, China

**Keywords:** maternal–fetal crosstalk, cardiovascular programming, intrauterine environment, developmental origins of health and disease

## Abstract

Cardiovascular disease (CVD) is the leading cause of morbidity and mortality worldwide, accounting for a substantial proportion of global deaths. Increasing evidence indicates that cardiovascular susceptibility is shaped during fetal development, where the intrauterine environment plays a critical role. Maternal–fetal crosstalk, mediated largely through placental function, coordinates the transfer of metabolic, endocrine, and immune signals that are essential for normal cardiac and vascular development. Disruptions in maternal physiology—including metabolic disorders, hypertensive conditions, inflammation, and environmental stress—can perturb this communication network and alter the intrauterine milieu. These changes induce persistent modifications in cardiomyocyte growth, endothelial function, and key regulatory pathways, thereby contributing to long-term cardiovascular risk. Emerging studies highlight that cardiovascular programming is governed by interconnected mechanisms involving epigenetic regulation, mitochondrial function, immune signaling, and intercellular communication. This review synthesizes current evidence on how maternal–fetal crosstalk shapes cardiovascular development beyond genetic determinants and provides an integrated framework linking early-life exposures to lifelong cardiovascular health.

## 1. Introduction

Cardiovascular disease (CVD) remains the leading cause of morbidity and mortality worldwide. According to the latest Global Burden of Disease study, CVD was responsible for approximately 19.2 million deaths globally in 2023 and remains a major contributor to disability and healthcare burden [1]. While traditionally linked to adult lifestyle and genetic predisposition, accumulating evidence indicates that cardiovascular risk is shaped much earlier in life [2]. Adverse environmental exposures during critical windows of fetal life can exert long-lasting effects on cardiac structure and vascular function, contributing to disease susceptibility across the lifespan [3].

These observations underpin the Developmental Origins of Health and Disease (DOHaD) framework, which recognizes early-life environmental exposures as pivotal determinants of long-term health [4,5]. Within this context, the intrauterine environment serves as the primary setting in which maternal factors influence fetal development. Under physiological conditions, maternal–fetal crosstalk is tightly coordinated through placental function, enabling the coordinated transfer of nutrients, hormones, and signaling molecules [6]. This dynamic regulatory system supports cardiac morphogenesis, vascular development, and the establishment of functional cardiovascular capacity. Disruptions in maternal physiology, including metabolic disorders, hypertensive complications, inflammation, and environmental stress, can interfere with maternal–fetal signaling and alter placental function [7,8,9,10] (Table 1). These disturbances reshape the intrauterine milieu, leading to sustained changes in cardiomyocyte growth, endothelial function, and regulatory pathways [11,12]. Such alterations may induce persistent reprogramming of cardiovascular structure and function, increasing the risk of hypertension, atherosclerosis, and heart failure later in life [7,13].

Advances in high-throughput omics, single-cell technologies, and in vivo imaging have provided new insights into the mechanisms underlying these processes [22,23]. Accumulating evidence suggests that cardiovascular programming is governed by interconnected pathways involving epigenetic regulation, mitochondrial function, immune signaling, and intercellular communication, often in a time-dependent manner during sensitive developmental windows [4].

In this review, we summarize current evidence on how maternal–fetal crosstalk shapes cardiovascular development beyond genetic determinants. We focus on maternal factors—including metabolic, endocrine, immune, and environmental influences—and discuss their roles in fetal cardiac and vascular programming. By integrating findings across systems and developmental stages, we aim to provide a comprehensive overview linking early-life exposures to lifelong cardiovascular health.

Literature search strategy

This narrative review was based on a targeted literature search of PubMed/MEDLINE and Web of Science covering studies published up to March 2026. The search strategy used combinations of keywords related to maternal–fetal crosstalk, cardiovascular programming, placenta, fetal cardiovascular development, metabolic disorders, hypertension, inflammation, immune regulation, endocrine signaling, and epigenetic mechanisms.

As a narrative review, no formal systematic review protocol or PRISMA framework was applied. Studies were selected based on relevance to the topic, with prioritization given to high-quality evidence, including large prospective human cohort studies, randomized controlled trials where available, and mechanistic animal studies providing causal insights into cardiovascular developmental programming.

## 2. Developmental Origins and Maternal–Fetal Crosstalk in Cardiovascular Programming

### 2.1. DOHaD Theory and Its Evolution in Cardiovascular Research

The DOHaD framework, initially proposed as the “Barker hypothesis,” posits that adverse environmental exposures during early development predispose individuals to chronic diseases later in life [2,5]. Early epidemiological studies linking low birth weight with increased cardiovascular risk provided the first evidence that intrauterine conditions exert long-term effects on health [81]. Subsequent clinical studies have reinforced this association, highlighting the importance of early-life environmental context in shaping disease susceptibility [7,62,82,83].

Over time, DOHaD has evolved from a descriptive epidemiological concept into a mechanistic framework integrating experimental and clinical evidence, with developmental plasticity as a central principle linking early-life exposures to long-term physiological outcomes [84]. Contemporary research refines this model by distinguishing between “instigator” mechanisms, representing upstream developmental insults such as nutritional imbalance or maternal disease that perturb developmental processes, and “effector” mechanisms, representing persistent, organ-specific structural and functional adaptations—including metabolic, endocrine, and epigenetic changes—that impose long-term physiological constraints and ultimately contribute to disease susceptibility [2]. Technological advances have further demonstrated that early environmental cues can be durably encoded through epigenetic and transcriptional modifications, operating within complex gene–environment interactions [4,85].

### 2.2. Maternal–Fetal Crosstalk as an Integrative Regulatory Axis

Within the DOHaD framework, maternal–fetal crosstalk has become recognized as a foundational axis connecting maternal environment to fetal cardiovascular development. This interaction extends beyond gestation to include the periconceptional period, during which parental nutritional, metabolic, and stress-related factors influence gamete quality and early embryogenesis [4].

Maternal metabolic, endocrine, and immune signals are integrated through the placenta to regulate fetal tissue patterning, vascular development, and cardiac morphogenesis [86]. This coordinated interaction has led to the concept of a “heart–placenta axis,” highlighting the tight coupling between placental function and cardiac development through shared pathways [87]. In addition, placental haemodynamics represent a critical regulatory layer, as abnormalities in uteroplacental perfusion—such as maternal and fetal vascular malperfusion—can impair spiral artery remodeling, increase vascular resistance, and restrict oxygen and nutrient delivery to the fetus, thereby influencing cardiovascular development [87]. At the molecular level, this crosstalk is mediated by multilayered signaling systems, including hormones, cytokines, and extracellular vesicles (EVs), which regulate fetal gene expression through epigenetic and post-transcriptional mechanisms [86]. Single-cell and epigenomic studies have increasingly shown that these signals function in a cell-type–dependent and temporally regulated manner, emphasizing the heterogeneity of placental and fetal cardiac responses to maternal signals [22,23]. Together, this integrative model underscores that cardiovascular programming arises from coordinated, multiscale interactions spanning molecular, cellular, and organ levels, rather than from isolated pathways.

## 3. Placenta as the Central Mediator of Maternal–Fetal Crosstalk

### 3.1. Placental Structure and Vascular Development

As a key component of the maternal–fetal interface, the placenta coordinates structural and vascular development with fetal cardiogenesis, governed by conserved pathways such as PlGF/VEGFR-1 [88]. Proper placental vascularization—trophoblast invasion, spiral artery remodeling, and villous branching—is essential for maintaining adequate fetal perfusion, and its disruption contributes to altered fetal hemodynamics and impaired cardiac morphogenesis [87]. Experimental evidence, including studies of GPR126 and CITED2, demonstrates that extra-cardiac defects in placental or early embryonic tissues can drive cardiac abnormalities [89,90]. Single-cell transcriptomic analyses reveal that placental endothelial and trophoblast subpopulations exhibit gene expression patterns paralleling developing cardiac and vascular cells [91]. In addition to transcriptional and cellular coordination, recent evidence highlights a crucial role for placental EVs in maternal-fetal communication. In mice, trophoblast-derived EVs released from differentiated placental cells promoted fetal cardiomyocyte proliferation and epicardial outgrowth [92]. These EVs carry miRNAs, proteins, and other bioactive molecules capable of modulating cardiomyocyte signaling and translational programs. For instance, pathological EVs derived from severe preeclampsia impair calcium handling in human iPSC-derived and murine cardiomyocytes [93], whereas EVs from normotensive placentae exert protective cardiovascular effects in hypertensive rats, reducing blood pressure progression and mitigating vascular and renal remodeling over 12 months [93,94,95].

### 3.2. Placental Dysfunction and Cardiovascular Outcomes

Placental dysfunction represents a fundamental pathological driver of adverse cardiovascular programming by disrupting the maternal-fetal signaling network [6]. Conditions such as preeclampsia, intrauterine growth restriction (IUGR), and maternal inflammation are characterized by impaired placental perfusion, oxidative stress, and dysregulated immune and endocrine signaling. These disturbances perturb fetal cardiac structure, vascular reactivity, and metabolic homeostasis [87].

Among these conditions, hypertensive disorders of pregnancy precipitate early-onset cardiovascular disease and cardiovascular mortality in offspring [7,96]. In parallel, placental insufficiency leading to IUGR has been shown to program persistent alterations in blood pressure regulation and vascular structure that extend into adulthood [97,98]. In addition, neutrophil-driven placental inflammation can impair placental barrier function, allowing maternal immune cells to infiltrate the fetal heart and disrupt cardiac macrophage composition and tissue architecture, thereby directly linking placental pathology to impaired cardiac development and long-term dysfunction [10]. These pathological processes are increasingly linked to cell-type-specific transcriptional and epigenetic reprogramming, with evidence highlighting a regulatory role for non-coding RNAs, particularly microRNAs, in mediating the long-term cardiovascular effects of placental dysfunction [99].

## 4. Maternal Metabolic Influences on Cardiovascular Development

### 4.1. Maternal Obesity and Lipid Metabolism

Maternal obesity stands as a primary driver of adverse cardiovascular programming, underpinned by dysregulated lipid metabolism and chronic low-grade inflammation. In obese pregnancies, elevated circulating free fatty acids and triglycerides cross the placenta, exposing the developing fetus to a lipotoxic intrauterine environment [100]. This disrupts cardiomyocyte proliferation and differentiation, impairs mitochondrial fatty acid oxidation, and promotes oxidative stress and inflammatory responses [101]. Similarly, maternal obesity is associated with reduced oxidative phosphorylation, diminished antioxidant capacity (e.g., downregulation of Sod1 and Gpx4), and alterations in developmental signaling pathways such as HIF-1α, ultimately compromising cardiac morphogenesis and maturation [102]. Increasing evidence further indicates that such metabolic changes may not be limited to a single generation. High-fat diet-induced lipotoxic cardiomyopathy has been shown to be transmitted across generations, potentially through epigenetic modifications including altered histone marks such as H3K27 trimethylation [103].

In line with these findings, evidence from both experimental and epidemiological studies supports the presence of structural and functional cardiac alterations in offspring. In human infants, maternal overweight, particularly in late gestation, predicts increased left ventricular wall thickness and chamber enlargement at birth and during the first year of life [14]. In minipigs, maternal high-fat diet exposure leads to greater left ventricular mass (+100%), larger chamber size (+100%), increased stroke volume (+75%), and early metabolic remodeling, including increased glucose uptake and glycogen accumulation at birth [14]. These offspring later develop myocardial insulin resistance, glycogen depletion, impaired fatty acid oxidation, and hyperdynamic systolic function, demonstrating persistence of cardiac abnormalities into adulthood [14].

Large-scale human cohort studies also quantify the long-term impact: compared with offspring of mothers with normal BMI, maternal obesity was associated with increased risk of cardiovascular disease from childhood to early adulthood, escalating in a dose-dependent manner across BMI categories (HR 1.10 for overweight; 1.16 for obesity grade I; 1.84 for obesity grade II; 2.51 for obesity grade III) [15]. Likewise, the Helsinki Birth Cohort Study reported that higher maternal BMI was linked to increased incidence of cardiovascular disease in offspring, with hazard ratios per kg/m^2^ of 1.022 (men) and 1.035 (women) [16]. These findings indicate that only a proportion of offspring develop clinically significant disease, but risk increases progressively with maternal obesity severity.

### 4.2. Gestational Diabetes and Glucose Signaling

Gestational diabetes mellitus is associated with an increased risk of cardiovascular disease in offspring, as demonstrated by large longitudinal cohort studies showing higher rates of early-onset conditions such as hypertension and heart failure from childhood into early adulthood [17]. Consistent with these long-term outcomes, clinical investigations have identified early alterations in cardiac structure and function in exposed offspring, including increased ventricular wall thickness, reduced ventricular dimensions, and decreased stroke volume, with the severity of these changes correlating with maternal glycemic control [13]. Notably, maternal glycemia may exert stage-dependent effects on cardiac development, as higher glucose levels in early pregnancy have also been associated with reduced left ventricular mass and end-diastolic volume in childhood, independent of conventional confounders [18].

These observations may be explained, at least in part, by the impact of excess maternal glucose on the fetal environment. Evidence from a prospective human cohort study of women with pregestational diabetes showed that abnormal fetal cardiac findings, including hypertrophic cardiomyopathy and cardiac dysfunction, were present in 60% of exposed fetuses and were associated with significantly elevated neonatal IGF-I concentrations, supporting a role for insulin/IGF signaling in cardiomyocyte hypertrophy and altered myocardial architecture [19]. In parallel, a human gestational diabetes cohort demonstrated increased oxidative stress, reduced antioxidant defense, and altered endothelial nitric oxide signaling in both maternal and cord blood, suggesting early vascular dysfunction in exposed offspring [20]. Importantly, the impact of diabetic pregnancy on offspring cardiac health may not be evident under normal physiological conditions but can become apparent under metabolic stress. In a rat model of maternal diabetes, offspring exhibited normal baseline cardiac function; however, exposure to a high-fat diet in adulthood resulted in cardiac hypertrophy, impaired myocardial function, and increased inflammatory cell infiltration, indicating enhanced susceptibility to later cardiovascular injury [21].

Recent studies demonstrate that maternal diabetes can also alter early cardiac development. Single-cell transcriptomic analyses in murine models reveal that maternal diabetes induces cell-type-specific transcriptional alterations in cardiac progenitor populations, particularly affecting second heart field cells and impairing cardiomyocyte differentiation, thereby increasing the risk of congenital heart defects [22]. Complementary multi-omics analyses further reveal that maternal metabolic disturbances can remodel the epigenomic landscape of discrete cardiac progenitor subsets, leading to aberrant lineage specification and developmental patterning defects [23].

### 4.3. Mitochondrial Dysfunction and Oxidative Stress

Mitochondrial function is essential for proper cardiac development, as it supports the high energetic demands of the developing heart while maintaining redox balance and cell survival. Growing evidence indicates that adverse intrauterine environments—including maternal metabolic disorders, hypoxia, and growth restriction—disrupt mitochondrial function in the placenta and fetal heart. For example, pregnancies complicated by IUGR exhibit reduced mitochondrial complex I activity and oxygen consumption in placental and neonatal tissues, highlighting early bioenergetic deficits in compromised fetal development [104]. Consistently, maternal diabetes and obesity impair mitochondrial respiration and autophagy in the offspring heart, with more pronounced and persistent effects observed in adult male offspring [105].

Disruption of mitochondrial homeostasis can impair cardiomyocyte differentiation and cardiac maturation through altered reactive oxygen species (ROS) signaling, dysregulated Ca^2+^ homeostasis, abnormal mitochondrial permeability transition pore (MPTP) activity, and disturbed mitochondrial dynamics [106]. In rat models, maternal exposure to 13–10.5% oxygen during gestation increased mitochondrial ROS emission, decreased respiratory capacity, and heightened susceptibility to ex vivo ischemia–reperfusion injury in adult offspring [107]. In addition, offspring with elevated mtDNA replication developed dilated cardiomyopathy and cardiac collapse shortly after birth, mediated by activation of the mitochondrial integrated stress response and ferroptosis pathways [108]. Maternal exposure to particulate matter in zebrafish disrupts mitochondrial biogenesis and increases mitochondrial ROS production in offspring cardiomyocytes, contributing to abnormal cardiac development [109].

Accumulating evidence suggests that targeting mitochondrial oxidative stress represents a promising strategy to mitigate developmental programming. Interventions that enhance antioxidant defenses, such as activation of NRF2 signaling or administration of mitochondria-targeted antioxidants (e.g., MitoQ) in animals, have been shown to reduce oxidative stress, improve mitochondrial function, and attenuate cardiac remodeling and vascular dysfunction in offspring exposed to adverse intrauterine conditions [110,111,112]. Consistently, antioxidant treatment in diabetic pregnancies alleviates pro-oxidant and pro-inflammatory signaling in the offspring heart, further supporting a causal role for oxidative stress in mediating programmed cardiovascular risk [113].

## 5. Endocrine Regulation in Maternal–Fetal Cardiovascular Crosstalk

### 5.1. Glucocorticoids

**Figure 1:**
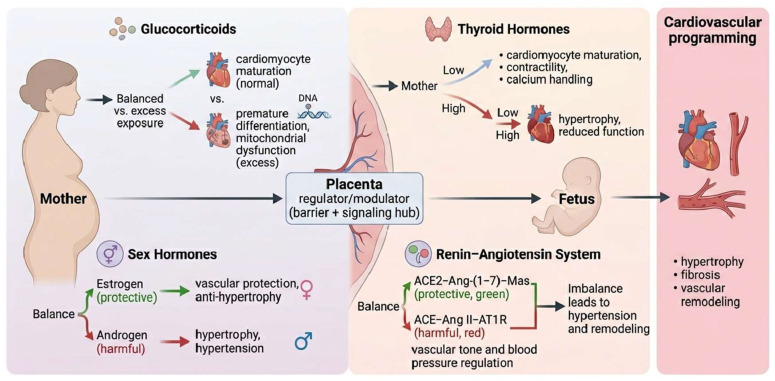
Endocrine signaling in maternal–fetal interaction.

Glucocorticoids serve as critical endocrine signals governing fetal organ maturation, particularly during late gestation, where they drive cardiomyocyte maturation by promoting cell cycle exit, functional differentiation, and metabolic adaptation. Physiological elevations in glucocorticoids facilitate the transition from proliferative to terminally differentiated cardiomyocytes, enhancing contractile function and preparing the heart for postnatal life. However, excessive exposure—arising from maternal stress, placental dysfunction, or exogenous corticosteroid administration—can disrupt this balance and lead to maladaptive cardiovascular programming [24] (Figure 1).

Experimental studies in animal models indicate that excess prenatal glucocorticoid exposure disrupts cardiac development and function. Antenatal glucocorticoid exposure induces premature cardiomyocyte differentiation, cardiac remodeling, and impairs mitochondrial function, leading to long-term cardiac vulnerability [25,26]. These effects are partially mediated by glucocorticoid receptor-dependent transcriptional regulation and epigenetic remodeling, including hypermethylation of the BMP4 promoter. This suppresses BMP4 expression, disrupts mitochondrial homeostasis, and heightens vulnerability to ischemia-induced injury, particularly under ischemia–reperfusion conditions [27,28]. Moreover, glucocorticoid-induced epigenetic alterations extend to vascular tissues, where disrupted DNA methylation and histone modifications impair endothelial nitric oxide signaling and promote vascular dysfunction [114,115].

However, evidence from randomized controlled trials and long-term follow-up studies in humans does not consistently support a detrimental effect of antenatal glucocorticoid exposure on cardiovascular outcomes. A landmark placebo-controlled trial with nearly 50-year follow-up found no significant differences in cardiovascular risk factors or major adverse cardiovascular events between individuals exposed to antenatal betamethasone and controls [29]. Correspondingly, exposure to repeat courses of antenatal glucocorticoids was not associated with increased cardiometabolic risk in childhood or early adulthood, with overall cardiovascular risk profiles remaining comparable between exposed and unexposed groups [30,31].

Four endocrine pathways (glucocorticoids, thyroid hormones, sex hormones, and renin–angiotensin system) are presented in separate panels, each illustrating directional signaling from maternal exposure → fetal cardiovascular effects → long-term offspring outcomes. Arrows indicate causal flow across compartments. Color coding denotes functional effects: green (protective) and red (deleterious). Glucocorticoids, thyroid hormones, and sex hormones regulate cardiomyocyte maturation and vascular development in a dose- and context-dependent manner, whereas RAS signaling reflects the balance between the ACE–Ang II–AT1R (injurious) and ACE2–Ang-(1–7)–Mas (protective) axes. Evidence is primarily derived from experimental models.

### 5.2. Thyroid Hormones

Thyroid hormones (TH) exert critical homeostatic control over cardiovascular development by regulating cardiac metabolism, contractility, and vascular maturation [32]. During fetal life, TH drives the transition of cardiomyocytes from proliferation to maturation, supporting structural and functional development of the fetal heart [33].

Experimental studies in animal models demonstrate that both insufficient and excessive maternal TH exposure during pregnancy can induce persistent alterations in cardiac structure and function. In transgenic mice, maternal hypothyroidism blunts postnatal cardiac maturation by disrupting myosin isoform switching and calcium-handling gene expression [34]. Similarly, rat models of fetal hypothyroidism revealed reprogrammed cardiac gene networks that ultimately reduce adult cardiac contractility [35]. Conversely, maternal hyperthyroidism or excess T3 exposure in rodents triggers cardiac hypertrophy, elevates heart rate, and dysregulates the cardiac renin–angiotensin system, thereby predisposing offspring to hypertension and impaired recovery from ischemic injury [11,32,36]. These effects may exhibit developmental stage- and sex-specific patterns, with late gestation identified as a particularly sensitive window for TH-mediated cardiac programming [32].

Consistent evidence has also been reported in human studies. Large population-based analyses from Danish registries indicate that maternal hypothyroidism elevated the risk of cardiovascular disease in offspring, including hypertension, arrhythmia, and myocardial infarction [37]. In addition, even subclinical alterations in maternal thyroid function are linked to higher blood pressure in offspring, suggesting that subtle hormonal imbalances during pregnancy may have long-term cardiovascular consequences [38].

### 5.3. Sex Hormones

Sex hormones modulate the cardiovascular system in a sex-dependent manner, with estrogens generally exerting protective effects and androgens contributing to adverse outcomes. Estrogen protects the cardiovascular system by maintaining vascular homeostasis and limiting adverse cardiac remodeling. An experimental study shows that estrogen protects against maternal high-fat diet-induced cardiac hypertrophy in female offspring, partly through regulation of angiotensin receptor signaling [39]. Notably, disruption of estrogen signaling during development impairs vascular formation and predisposes offspring to hypertension, an effect that can be reversed by estradiol supplementation [40]. Consistent with these findings, estrogen also attenuates programmed hypertensive responses in offspring exposed to maternal gestational hypertension [41].

In contrast, excess androgen exposure during critical developmental windows drives long-term cardiovascular dysfunction. Prenatal androgen excess induces cardiac hypertrophy and activates pro-hypertrophic signaling pathways, while simultaneously suppressing cardiomyocyte proliferation through cell cycle arrest mechanisms [42,43,44]. Besides, androgens promote vascular dysfunction and hypertension via androgen receptor-mediated upregulation of vasoconstrictive pathways and epigenetic suppression of protective estrogen signaling [45,46]. Moreover, maternal hyperandrogenic conditions, such as polycystic ovary syndrome, evoke elevated blood pressure and cardiac remodeling in offspring [47,48].

### 5.4. Renin–Angiotensin System Signaling

The renin–angiotensin system (RAS) regulates cardiovascular homeostasis and is widely expressed in the placenta and developing cardiovascular, renal, and neural systems during fetal life [116]. During early life, coordinated activity of both the classical angiotensin-converting enzyme–angiotensin II–angiotensin II type 1 receptor (ACE–Ang II–AT1R) axis and the counter-regulatory ACE2–Ang-(1–7)–Mas receptor axis is essential to maintain vascular tone, organ growth, and blood pressure control. Disruption of this balance stands as a fundamental mechanism underlying cardiovascular programming, as adverse intrauterine environments preferentially enhance the Ang II–AT1R pathway while suppressing protective Ang-(1–7) signaling, thereby predisposing offspring to hypertension and cardiometabolic disorders [117].

A wide range of prenatal insults—including maternal hypertension, preeclampsia, undernutrition, and altered salt or hormonal environments—has been shown to induce persistent alterations in RAS activity. For instance, prenatal exposure to elevated Ang II in a rat model directly increased blood pressure and altered cardiac and renal development in offspring [49]. Similarly, animal studies demonstrated that maternal high-salt intake reshapes the expression of RAS components (e.g., ACE, AT1, AT2, and Mas receptors), driving vascular remodeling, oxidative stress, and long-term susceptibility to hypertension [50,51]. Evidence from a human cohort study showed that neonates exposed to preeclampsia displayed altered coronary artery dimensions and dysregulated angiotensin receptor signaling, indicating that placental dysfunction can influence perinatal cardiovascular development [52]. Furthermore, infusion of agonistic AT1 receptor autoantibodies (AT1-AA) into pregnant rats elevated blood pressure and induced endothelial dysfunction in adult offspring [53].

These alterations in RAS signaling are accompanied by oxidative stress, inflammation, and vascular remodeling. For example, maternal undernutrition in rats upregulated vascular ACE and AT1 receptor expression, exacerbating oxidative damage and promoting arterial remodeling in adult offspring [54]. Targeting the RAS during developmental windows has shown beneficial effects in experimental models. In hypertensive rats, maternal administration of Ang-(1–7) or the ACE2 activator during gestation attenuated offspring hypertension, improved vascular function, and reduced cardiac remodeling in adulthood [55].

## 6. Immune and Inflammatory Mechanisms in Cardiovascular Development

### 6.1. Maternal Immune Tolerance

**Figure 2:**
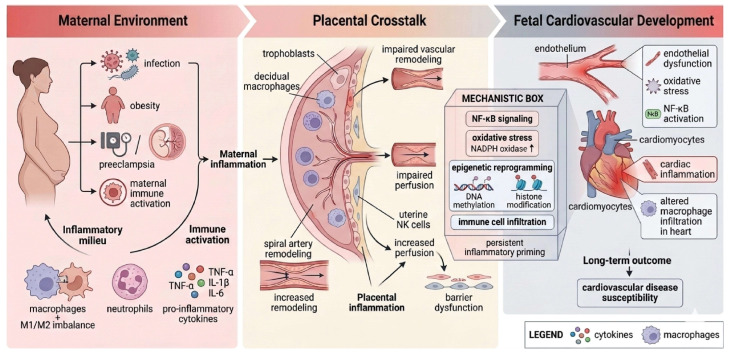
Immune and inflammatory mechanisms in maternal–fetal cardiovascular programming.

Successful pregnancy requires a finely tuned maternal immune environment that promotes fetal tolerance while preserving host defense. This balance supports not only placental formation but also fetal cardiovascular development. At the maternal–fetal interface, coordinated interactions between extravillous trophoblasts and maternal immune cells establish a tolerogenic and pro-angiogenic microenvironment that supports spiral artery remodeling and adequate placental perfusion [118]. Uterine immune populations—particularly macrophages and decidual natural killer cells—govern vascular remodeling and influence fetal cardiac loading conditions [119] (Figure 2).

Disruption of maternal immune tolerance—such as inflammation, metabolic disorders, or immune activation—can impair placental vascular development and alter fetal hemodynamics, thereby altering the trajectory of cardiovascular development and increasing susceptibility to cardiac dysfunction later in life, particularly under conditions of secondary stress [120]. Recent single-cell transcriptomic analyses of human fetal hearts exposed to maternal anti-SSA/Ro autoimmunity have revealed heightened and heterogeneous interferon responses across multiple cardiac cell types, along with increased expression of extracellular matrix-related genes implicated in fibrosis, providing evidence that maternal immune dysregulation can reshape the fetal cardiac microenvironment at the molecular level [60]. In this context, maternal immune activation has been linked to elevated pro-inflammatory cytokine exposure in utero and subsequent immune dysregulation in offspring, processes potentially mediated by epigenetic modifications [61].

### 6.2. Innate Immunity and Macrophage Heterogeneity

Innate immune cells, particularly macrophages, serve as indispensable orchestrators of placental function and fetal cardiovascular development. In the developing heart, resident macrophages contribute to angiogenesis, extracellular matrix remodeling, and tissue maturation [121]. Recent single-cell RNA sequencing studies have revealed remarkable heterogeneity among macrophage populations during human development, identifying specialized subsets such as pro-angiogenic and microglia-like macrophages that localize to perivascular regions and participate in organogenesis, including cardiac development [122].

This functional heterogeneity is shaped by macrophage origin, with embryonically derived CCR2^−^ resident macrophages supporting tissue development and repair, while monocyte-derived CCR2^+^ macrophages are more closely associated with inflammatory responses and tissue injury [123]. Consistent with this, fate-mapping and single-cell analyses in vascular tissues demonstrate that yolk sac-derived macrophages establish long-lived, self-renewing populations with homeostatic and regenerative roles, whereas bone marrow-derived monocytes are preferentially recruited during inflammation and drive distinct transcriptional programs governing tissue injury and repair [124]. Maternal environmental factors can impair macrophage polarization and function. For instance, maternal hypercholesterolemia promotes a shift toward pro-inflammatory M1 polarization in offspring macrophages through epigenetic reprogramming, thereby increasing susceptibility to atherosclerosis [125]. Similarly, maternal obesity induces early-life immunometabolic alterations in bone marrow-derived myeloid cells, including disrupted lipid metabolism and altered gene expression in pathways related to macrophage activation [126,127].

At the maternal–fetal interface, decidual macrophages are central mediators of placental inflammation. In mouse models, inflammatory activation of placental macrophage populations increases production of TNF-α, IL-1β, and IL-6, which can compromise placental vascular homeostasis and fetal exposure to inflammatory signals [56]. Experimental depletion or modulation of maternal immune cells in pregnant mice further demonstrates that altered decidual immune composition disrupts placental structure and inflammatory signaling, impairing fetal development [10].

Maternal immune activation (infection, obesity, preeclampsia) induces systemic inflammation characterized by macrophage imbalance, neutrophil activation, and elevated cytokines (TNF-α, IL-1β, IL-6), which are transmitted via the placenta. This disrupts trophoblast function, spiral artery remodeling, and fetal exposure to inflammatory signals. In the fetus, NF-κB activation, oxidative stress, and endothelial dysfunction drive maladaptive cardiovascular development and long-term disease susceptibility. Epigenetic reprogramming contributes to persistent inflammatory priming and potential transgenerational risk transmission. Evidence derives from experimental models and human studies.

### 6.3. Inflammation and Endothelial Dysfunction

Maternal inflammation contributes to fetal vascular dysfunction and subsequent cardiovascular risk. Conditions such as infection, obesity, and preeclampsia expose the fetus to a pro-inflammatory and oxidative intrauterine milieu, leading to early disruption of cardiovascular homeostasis [57].

A key mechanistic cascade involves macrophage infiltration into the decidua, which enhances placental production of pro-inflammatory cytokines. In a murine model of intra-amniotic LPS exposure, the activation of placental macrophages increased TNF-α, IL-1β, and IL-6 expression in the placenta, which was accompanied by fetal cardiac hemodynamic impairment despite minimal direct fetal inflammation [56]. Consistently, a neutrophil-driven placental inflammation model in mice demonstrated that maternal immune activation disrupts placental barrier integrity, allowing maternal inflammatory monocytes to infiltrate fetal tissues. These cells subsequently alter embryonic cardiac macrophage composition and disrupt cardiac tissue architecture, resulting in persistent postnatal cardiac dysfunction [10]. Mechanistically, maternal inflammatory exposure activates NF-κB signaling and amplifies pro-inflammatory cytokine production in cardiovascular tissues, establishing a sustained, primed inflammatory state [58,59]. This is accompanied by dysregulated oxidative stress responses, characterized by enhanced NADPH oxidase activity and impaired antioxidant capacity, which together form a feed-forward loop that exacerbates vascular injury [58,59]. Importantly, the effects of maternal inflammation may persist beyond a single generation. Prenatal inflammatory exposure can induce long-lasting epigenetic modifications, including DNA methylation and histone modifications in vascular and germline cells, thereby contributing to persistent endothelial dysfunction and transgenerational transmission of hypertension risk [128,129].

## 7. Environmental and Lifestyle Determinants of Cardiovascular Risk

### 7.1. Environmental Pollutants and Endocrine Disruption

Exposure to environmental pollutants—including particulate matter (PM2.5), heavy metals, and endocrine-disrupting chemicals (EDCs) during pregnancy can cross the placental barrier, impair placental function, and trigger oxidative stress, inflammation, and endocrine imbalance [62,63]. For example, a large population-based case–control study involving more than 1.4 million births in China reported that each 10 μg/m^3^ increase in maternal PM2.5 exposure during the periconceptional period was associated with a 2% increase in CHD risk (OR = 1.02), with septal defects showing higher vulnerability (OR = 1.04), highlighting early developmental windows as periods of increased susceptibility [62]. Experimental and clinical studies further indicate that prenatal exposure to EDCs, such as bisphenol A, disrupts hormonal homeostasis and contributes to adverse cardiovascular outcomes across the lifespan, partly through epigenetic modifications affecting cardiac structure, extracellular matrix remodeling, and energy metabolism [63,64]. Corroborating these observations, prenatal environmental exposures can exert long-lasting effects on cardiovascular function through epigenetic programming. For instance, antenatal nicotine exposure reprograms vascular function via RNA epigenetic mechanisms, whereby upregulation of the m6A demethylase FTO enhances NOX2-dependent oxidative stress and elevates blood pressure in offspring [65].

### 7.2. Maternal Stress and Neuroendocrine Signaling

Maternal stress influences fetal cardiovascular development through neuroendocrine perturbations, principally via activation of the hypothalamic–pituitary–adrenal (HPA) axis and subsequent alterations in glucocorticoid signaling.

Large population-based cohort studies in humans suggest that overall maternal bereavement during or shortly before pregnancy is not consistently associated with ischemic heart disease or stroke in offspring; however, exposure to severe stressors, such as the death of a partner or older child, particularly during the third trimester, is linked to substantially increased risk of heart failure and ischemic heart disease in offspring (HR 1.47–2.77) [66,67]. Additional human cohort data indicate a modest association between prenatal stress and offspring cardiovascular disease or hypertension, although these effects are attenuated when controlling for familial factors [8]. Animal studies provide complementary mechanistic evidence. Pre-gestational chronic mild stress models extending through pregnancy and lactation in rodents demonstrate that sustained maternal depressive-like states result in persistent hormonal and behavioral alterations, along with cardiac structural and functional changes in offspring [68]. In addition, maternal circadian disruption during pregnancy, studied in rodents, impairs placental function and increases susceptibility to metabolic and cardiac dysregulation in offspring, particularly under dietary challenges [69]. Pharmacological modulation of maternal stress pathways also affects fetal cardiac development. In rodent models, selective serotonin reuptake inhibitors (SSRIs) cross the placenta, decrease cardiomyocyte proliferation, disrupt calcium handling, and alter serotonin-related gene and microRNA expression in the developing heart, with functional consequences persisting into adulthood [70].

### 7.3. Maternal Nutrition and Metabolic Programming

Both undernutrition and overnutrition can disrupt metabolic–vascular coupling, predisposing offspring to long-term cardiovascular dysfunction. Deficiencies in key micronutrients—such as folate, iron, and vitamin D—have been consistently linked to impaired angiogenesis, endothelial dysfunction, and aberrant cardiac morphogenesis. For instance, a prospective case–control study in China reported that mothers with periconception red blood cell folate ≥906 nmol/L had a 39% lower risk of congenital heart disease in their children compared with those with lower folate levels (adjusted OR 0.61, 95% CI 0.40–0.93), and a Mendelian randomization analysis suggested a 25% risk reduction per 100-nmol/L increase in maternal RBC folate (OR 0.75, 95% CI 0.61–0.92) [71]. Moreover, accumulating evidence suggests that both insufficient and excessive folate levels may adversely affect cardiac development through interactions within the one-carbon metabolic network, including vitamin B12 status and homocysteine levels, which are implicated in long-term cardiovascular and neuroendocrine programming in offspring [71,72]. Similarly, animal studies have shown that maternal iron deficiency induces severe cardiovascular malformations in offspring, potentially via dysregulated retinoic acid signaling pathways [73]. Experimental studies suggest that gestational vitamin D deficiency disrupts cardiomyocyte maturation and promotes postnatal cardiac remodeling, characterized by delayed maturation and compensatory hypertrophy that may predispose to long-term dysfunction [74,75].

Excessive caloric intake and imbalanced maternal diets, particularly those enriched in saturated fatty acids or fructose, can exacerbate oxidative stress, inflammation, and metabolic dysregulation, thereby increasing cardiovascular risk across generations. Evidence from murine models demonstrates that maternal high-fat diet exposure during gestation and lactation programs adverse cardiovascular and metabolic phenotypes in offspring, including cardiac hypertrophy, hypertension, insulin resistance, and pathological cardiac remodeling [76,77]. Specifically, lipid metabolites such as elevated palmitic acid impair cardiac development via MARS-mediated lysine homocysteinylation of critical cardiac transcription factors [78]. Likewise, mouse studies have shown that maternal high-fructose intake induces multigenerational activation of the renin–angiotensin–aldosterone system, elevating blood pressure, inflammation, and cardiac fibrosis in offspring [79]. Recent advances in metabolomics and epigenomic profiling further reveal that maternal diet shapes the fetal epigenetic landscape, including DNA methylation and chromatin modifications, thereby establishing long-lasting alterations in metabolic phenotype and vascular function. For instance, nutrient-sensitive epigenetic regulators, such as TET2-mediated DNA demethylation and metabolic intermediates like carbamoyl phosphate, have been implicated in cardiogenic gene regulation and CHD susceptibility [80].

## 8. Epigenetic Regulation of Cardiovascular Programming

**Figure 3:**
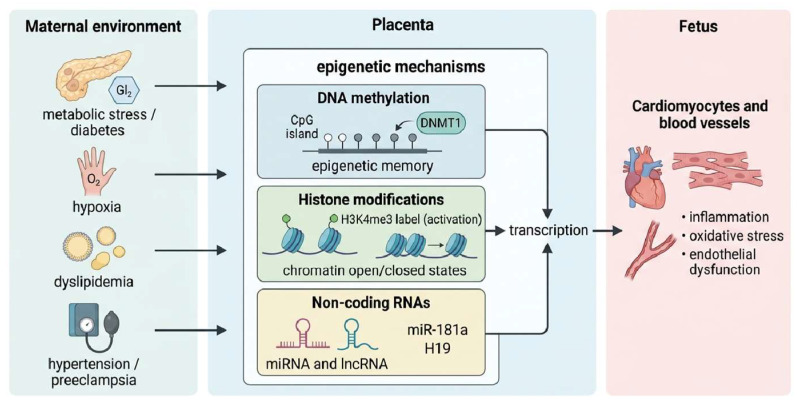
Epigenetic mechanisms underlying maternal–fetal cardiovascular programming.

Epigenetic regulation represents a central mechanism through which maternal environmental cues are translated into long-term changes in the fetal cardiovascular phenotype. As a core component of developmental plasticity, epigenetic modifications enable the fetus to adapt to intrauterine conditions without altering the DNA sequence, although such adaptations may prove maladaptive later in life [130]. DNA methylation, histone modifications, and non-coding RNAs (ncRNAs) collectively shape chromatin architecture and gene expression in a dynamic and context-dependent manner (Figure 3).

Accumulating evidence demonstrates that adverse intrauterine exposures—including metabolic stress, hypoxia, dyslipidemia and hypertensive disorders of pregnancy—induce locus-specific DNA methylation changes in genes involved in vascular function and cardiac development. In a human epigenome-wide association study of 388 Pima Indian offspring exposed to maternal diabetes in utero, differential DNA methylation was detected at multiple loci involved in metabolic regulation, with some methylation signatures predicting future diabetes risk and increased adiposity, both established cardiovascular risk factors [131]. Similarly, analysis of 78 human fetal aortas showed that maternal hypercholesterolemia was associated with increased methylation and reduced expression of SREBP2, accompanied by larger fetal aortic lesions, providing direct evidence that maternal lipid status influences fetal vascular development through epigenetic mechanisms [132]. In another large human meta-analysis involving over 5000 mother–offspring pairs, hypertensive disorders of pregnancy were associated with differential methylation at multiple CpG sites in cord blood, with several of these epigenetic signatures persisting into adolescence, suggesting long-term biological effects [133]. Recent investigations further suggest that adverse intrauterine environments can establish a persistent epigenetic “memory” that links early-life exposure to adult disease susceptibility. For example, in animal models, maternal diabetes induces DNMT1-dependent reprogramming of hematopoietic progenitors, while maternal Western-type diet promotes AP-1-associated chromatin remodeling and inflammatory memory in endothelial cells, collectively predisposing offspring to cardiometabolic and atherosclerotic disease [9,134]. Prenatal hypoxia in rats increases CpG methylation at the PKCε promoter in fetal hearts, leading to sustained PKCε repression and heightened susceptibility to ischemic cardiac injury, whereas perinatal nicotine exposure in rodents alters DNA methylation of the AgtR1 gene in carotid bodies, enhancing sympathetic activation and predisposing offspring to hypertension [135,136].

Histone modifications contribute to epigenetic programming by regulating chromatin accessibility. In humans, gestational diabetes is associated with persistent enrichment of H3K4me3 at inflammatory gene promoters in offspring blood cells, indicating sustained epigenetic activation [129]. In animal models, Brg1–HDAC complexes regulate embryonic cardiac gene programs, including the α-MHC/β-MHC switch, linking chromatin remodeling to cardiac growth and hypertrophy in mice [137]. Maternal high-fat diet during pregnancy in mice induces altered chromatin accessibility and reduced 5hmC in fetal mouse hearts, leading to developmental cardiac defects [138]. In rats, maternal metabolic stress and nicotine exposure also reprogram histone marks and DNA methylation in cardiac regulatory genes, contributing to long-term susceptibility to dysfunction [12,139].

Recent high-resolution and cell-specific analyses reveal that these epigenetic modifications are highly dynamic and cell-type-dependent. For instance, gestational diabetes induces distinct DNA methylation and transcriptional alterations in different fetoplacental endothelial cell subtypes, leading to changes in cytoskeletal organization and barrier function [140].

Adverse maternal environments (metabolic stress, hypoxia, dyslipidemia, and hypertensive disorders) are transmitted via the placenta to the fetus and reprogram cardiovascular development through epigenetic mechanisms. These include DNA methylation (DNMT1-mediated CpG modifications and epigenetic memory), histone modifications (e.g., H3K4me3), and non-coding RNAs (miR-181a, H19), which collectively regulate transcriptional programs. These changes induce persistent inflammatory activation, oxidative stress, and endothelial dysfunction in fetal cardiovascular tissues, promoting long-term disease susceptibility. Arrows indicate maternal–placental–fetal directionality; epigenetic modules are shown in the central panel. Evidence derives from experimental models and human epigenome-wide association studies.

## 9. Sex Differences in Cardiovascular Outcomes

Males and females exhibit distinct cardiovascular disease risks, with males generally showing higher susceptibility to hypertension and cardiac remodeling, while females may be more prone to vascular dysfunction under certain prenatal insults. Accumulating data suggest that these differences are established, at least in part, through sex-dependent placental adaptations. Maternal metabolic disturbances induce sexually dimorphic changes in placental function, including autophagy activity, immune cell composition, and lipid transport, which may differentially shape cardiometabolic risk in male and female offspring [141,142]. In addition, sex-biased epigenetic regulation in the placenta, particularly in DNA methylation patterns and imprinted gene regions, provides a molecular basis for divergent developmental trajectories and long-term disease susceptibility [143]. These early differences are further reflected in offspring cardiovascular phenotypes. Prenatal insults such as hypoxia and parental obesity have been shown to program vascular function, cardiac remodeling, and blood pressure regulation in a sex-dependent manner, with some studies reporting greater effects in males and others demonstrating more pronounced vascular alterations in females [144,145,146].

## 10. Translational and Clinical Implications

### 10.1. Biomarkers for Early Cardiovascular Risk Prediction

The identification of reliable biomarkers for early cardiovascular risk prediction is a key priority in translating DOHaD concepts into clinical practice. Increasing evidence suggests that placenta-derived signals—including ncRNAs, angiogenic factors, and EVs—serve as sensitive indicators of intrauterine environmental perturbations and early cardiovascular programming [93,147]. EVs are increasingly recognized as important mediators of maternal–fetal communication, capable of transporting diverse bioactive molecules such as miRNAs, peptides, and lipids, thereby reflecting the status of both mother and fetus [93].

Among these, circulating microRNAs (miRNAs) represent particularly promising biomarkers due to their stability and regulatory roles in vascular and metabolic pathways. Altered miRNA expression profiles have been consistently observed in both mothers and offspring exposed to hypertensive pregnancy disorders such as preeclampsia, with certain changes persisting for years postpartum, suggesting sustained involvement in cardiovascular disease susceptibility [147]. Furthermore, endothelial-specific miRNA signatures (e.g., elevated miR-146a) identified at birth are associated with impaired angiogenesis, inflammatory signaling, and altered microvascular development, and can predict early postnatal vascular phenotypes [148]. In addition, circulating cell-free miRNAs in maternal blood have shown potential as noninvasive biomarkers for early detection of fetal cardiac abnormalities, including congenital heart disease, highlighting their diagnostic and mechanistic relevance [149].

Beyond ncRNAs, placental angiogenic factors such as placental growth factor (PGF) and soluble fms-like tyrosine kinase-1 (sFlt-1) also demonstrate predictive value. Subtle alterations in maternal blood pressure during early pregnancy—preceding clinically overt hypertensive disorders—are associated with offspring blood pressure trajectories, with PGF acting as a critical mediator linking maternal vascular status to offspring cardiovascular outcomes [150]. These findings underscore the importance of integrating maternal hemodynamic and placental biomarker data for early risk identification. Moreover, first-trimester maternal plasma proteomic profiling combined with machine learning has demonstrated high accuracy in detecting fetal congenital heart disease, directly uncovering dysregulated pathways governing metabolism, extracellular matrix remodeling, and cardiac function [151].

### 10.2. Preventive Strategies During Pregnancy

Preventive interventions targeting the intrauterine environment represent a promising strategy to mitigate long-term cardiovascular risk. Optimizing maternal metabolic health—through dietary regulation, weight management, and glycemic control—has been shown to improve placental function and fetal cardiovascular outcomes [152]. In particular, maternal nutrition plays a pivotal role in developmental programming. For example, in a rat model of maternal protein restriction, folate supplementation improved vascular function and prevented offspring hypertension [153]. Consistent with these experimental findings, a large human case–control study demonstrated that first-trimester folic acid supplementation was associated with a lower risk of congenital heart defects, particularly severe phenotypes [154]. Conversely, findings from a mouse model indicate that excessive supplementation may be detrimental; maternal folic acid over-supplementation compromised cardiac function in offspring through activation of DNA methylation and oxidative stress pathways [155].

Maternal dietary composition also exerts long-term effects on offspring cardiovascular development. In a mouse model, maternal high-fiber intake effectively reshaped the offspring’s cardiac immune landscape and promoted anti-fibrotic and anti-inflammatory transcriptional profiles, potentially via microbiota-derived short-chain fatty acids [156]. In contrast, large-scale human data suggest that protein–calorie supplementation alone may have limited long-term cardiovascular benefits, underscoring the complexity of nutritional interventions [157].

Importantly, these findings highlight that critical programming events occur early in development, including the preconception period. Accordingly, lifestyle optimization—such as balanced nutrition and physical activity—orchestrates maternal physiological adaptations to support healthy fetal development [156,158,159]. Together, this supports a lifecourse-based approach to reducing cardiovascular risk.

### 10.3. Therapeutic Targeting of Maternal–Fetal Pathways

Targeting specific maternal–fetal signaling pathways offers novel therapeutic opportunities to modulate developmental programming. Interventions aimed at improving placental function, mitigating oxidative stress, and regulating inflammatory signaling are under active investigation, mostly in preclinical stages. For example, while low-dose aspirin has an established clinical role in pregnancies at high risk for hypertensive disorders, other pharmacological agents, such as antioxidants, remain largely experimental [111,160]. Maternal administration of the xanthine oxidase inhibitor allopurinol during hypoxic pregnancy has been shown to restore cardiac function and normalize molecular markers of myocardial stress in adult rat offspring, highlighting oxidative stress as a critical target [161]. These findings are primarily based on rat models and provide mechanistic insight into oxidative stress-related programming. Similarly, prenatal sildenafil administration in fetal growth restriction models improves vascular reactivity and attenuates the progression of hypertension in rat offspring [162]. However, randomized human trials have raised severe neonatal safety concerns, leaving its clinical use strictly investigational [162].

Beyond oxidative and vascular pathways, cholesterol and lipid metabolism constitute vital therapeutic targets. In C57BL/6 mouse models, maternal treatment with pravastatin in hypercholesterolemic pregnancies reduces offspring blood pressure and restores healthy lipid profiles into adulthood [163]. Similar protective effects against offspring atherogenesis have been observed in LDL-receptor-deficient mice and New Zealand White rabbits following maternal immunization against oxidized LDL [164]. Nonetheless, statins remain generally contraindicated during human pregnancy, and current evidence for developmental benefits remains preclinical. Emerging studies also highlight the promise of epigenetic regulation. For example, prenatal inhibition of the histone demethylase LSD1 rescues defective cardiac differentiation and prevents cardiomyopathy in Lmna-mutant mouse models, demonstrating the therapeutic potential of targeting epigenetic modifiers in utero [165].

Furthermore, increasing interest has focused on microbiota-mediated and gut–organ axis signaling as novel therapeutic targets. Modulating maternal gut microbiota, as demonstrated by captopril-induced changes in the gut–brain axis in hypertensive rat models, may represent an indirect yet effective strategy to safeguard offspring cardiovascular health [166]. Additionally, advances in nanotechnology raise the possibility of precisely targeting placental or fetal signaling pathways [167,168]. These approaches are still in early development and have not yet reached clinical validation. Although these precision-medicine approaches remain at the experimental stage, they collectively highlight the potential of targeting maternal–fetal crosstalk to prevent and treat cardiovascular disease at its developmental origins.

## 11. Discussion

This review establishes maternal–fetal crosstalk as the foundational framework for understanding cardiovascular programming, illustrating how early-life environmental cues are integrated to shape long-term cardiovascular health. Increasing evidence suggests that the intrauterine period represents a critical window for intervention, during which modulation of maternal metabolism, inflammation, or placental function may redirect disease trajectories before clinical manifestation [10,17]. In this context, strategies—such as nutritional optimization, antioxidant therapy, and targeted pathway interventions—offer promising avenues for early prevention of cardiovascular disease [112,157].

Despite these advances, several limitations must be addressed. First, much of the existing mechanistic evidence derives from animal models, leaving its translatability to human pregnancy constrained by species-specific variations in placental architecture, gestational kinetics, and cardiovascular physiology. Second, maternal prenatal exposures rarely occur in isolation; metabolic disturbances, inflammation, hormonal shifts, and environmental stressors frequently coexist and interact, making it exceptionally challenging to disentangle their individual contributions to offspring outcomes. Consequently, delineating whether downstream effects stem from the direct placental transfer of maternal factors or from secondary, autonomous epigenetic and transcriptional reprogramming within the fetus remains difficult. Furthermore, the current scarcity of large-scale, lifelong prospective human cohorts limits our capacity to establish definitive causal links between prenatal molecular alterations and adult cardiovascular disease.

To bridge these gaps, future research could focus on deciphering the precise causal pathways mediating maternal–fetal communication, defining exact developmental windows of susceptibility, and validating robust, non-invasive biomarkers for early risk stratification. Integrating longitudinal clinical cohorts with single-cell multi-omics, spatial transcriptomics, and computational modeling will refine our mechanistic understanding and accelerate the development of safe, targeted interventions during pregnancy.

## 12. Conclusions

Maternal–fetal crosstalk serves as the primary mechanism linking the intrauterine environment to lifelong cardiovascular health. Through the placenta, maternal metabolic, endocrine, immune, and environmental signals converge to orchestrate fetal cardiac and vascular development, leaving persistent biological signatures that dictate disease susceptibility later in life. Current evidence underscores that cardiovascular programming arises from the systemic integration of multiple interacting networks rather than isolated environmental insults. A deeper understanding of these multifaceted mechanisms and how they operate is essential to identify high-risk pregnancies and pioneer effective, early-life preventive strategies.

## Figures and Tables

**Table 1:** Summary of maternal exposures, placental mediators, and fetal cardiovascular outcomes in developmental programming of cardiovascular disease.

Maternal Exposure	Placental Mediator	Fetal Cardiovascular Effect	Evidence Type	Clinical Relevance
Maternal obesity	Elevated free fatty acids, triglycerides; placental metabolic disruption	Impaired cardiomyocyte proliferation/maturation; increased LV mass and chamber enlargement; early metabolic remodeling	Human cohort [14,15,16]; Animal: minipigs [14]	Increased risk of cardiovascular disease in offspring, dose-dependent with maternal BMI
Gestational diabetes	Maternal hyperglycemia, insulin/IGF signaling, oxidative stress	Cardiac hypertrophy; reduced stroke volume; altered ventricular wall thickness; congenital heart defects	Human cohort [17,18,19,20]; Animal: rat models [21]; Murine models [22,23]	Early-onset hypertension, heart failure; long-term susceptibility to metabolic stress
Excess glucocorticoids	Placental glucocorticoid transfer	Premature cardiomyocyte differentiation; cardiac remodeling; mitochondrial dysfunction	Animal: rodent studies [24,25,26,27,28]; Human RCT [29,30,31]	Potential long-term cardiac vulnerability
Thyroid hormone imbalance	TH levels crossing placenta	Cardiac hypertrophy; altered contractility; impaired recovery from ischemic injury	Animal: mice, rat [32,33,34,35,36]; Human: [37,38]	Increased risk of hypertension, arrhythmia, myocardial infarction
Excess androgens/low estrogens	Placental hormone signaling	Cardiac hypertrophy; vascular dysfunction; hypertension; impaired cardiomyocyte proliferation	Animal: rodent studies [39,40,41,42,43,44,45,46,47,48]	Long-term cardiovascular dysfunction
Dysregulated RAS signaling	Altered ACE/Ang II/AT1R/Ang-(1–7)/Mas receptor expression	Hypertension; vascular remodeling; oxidative stress; cardiac remodeling	Animal: rat [49,50,51,52,53,54,55]; Human cohorts [52]	Predisposition to hypertension and cardiometabolic disorders
Maternal inflammation/immune activation	Placental macrophages, cytokines (TNF-α, IL-1β, IL-6), NF-κB signaling	Fetal cardiac hemodynamic impairment; altered embryonic cardiac macrophage composition; fibrosis	Animal: murine models [10,56,57,58,59]; Human [60,61]	Persistent postnatal cardiac dysfunction; transgenerational endothelial dysfunction
Environmental pollutants	Placental oxidative stress, inflammation, endocrine disruption	Abnormal cardiac development; extracellular matrix remodeling; altered energy metabolism	Human study [62,63]; Animal models [63,64,65]	Increased risk of congenital heart defects; epigenetic programming of cardiovascular risk
Neuroendocrine disruption	HPA axis activation; glucocorticoid signaling; SSRIs	Altered cardiomyocyte proliferation; disrupted calcium handling; serotonin pathway modulation	Human cohort [8,66,67]; Animal rodent studies [68,69,70]	Increased risk of heart failure, ischemic heart disease; long-term cardiac dysfunction
Maternal nutrition	Folate, iron, vitamin D, lipid metabolites	Impaired angiogenesis; endothelial dysfunction; cardiac hypertrophy and remodeling	Human cohort [71,72]; Animal models [73,74,75,76,77,78,79,80]	Modulation of congenital heart defect risk; long-term vascular and metabolic programming

Evidence is derived from human cohort studies, animal experimental models, and mechanistic studies, with prioritization given to higher-level clinical and translational evidence where available. Abbreviations: LV, left ventricle; ROS, reactive oxygen species; RAS, renin–angiotensin system; IGF, insulin-like growth factor; EDCs, endocrine-disrupting chemicals; CHD, congenital heart defects.

## Data Availability

No data were created or analyzed in this study.

## Abbreviations

The following abbreviations are used in this manuscript:

ACE	Angiotensin-Converting Enzyme
Ang II	Angiotensin II
AT1R	Angiotensin I Receptor
AT2R	Angiotensin II Receptor
CHD	Congenital Heart Disease
CVD	Cardiovascular Disease
DOHaD	Developmental Origins of Health and Disease
EDCs	Endocrine-Disrupting Chemicals
EVs	Extracellular Vesicles
IUGR	Intrauterine Growth Restriction
ncRNAs	Non-coding RNAs
RAS	Renin–Angiotensin System
TH	Thyroid Hormones
